# Studies in the Psychology of Sex

**Published:** 1900-03

**Authors:** 


**Studies in the Psychology of Sex.** By HAVELOCK ELLIS. Vol. I. —*Sexual Inversion.* Pp. xvii., 204. London: The University Press. 1897. Vol. II.—*The Evolution of Modesty. The Phenomena of Sexual Periodicity. Auto-Erotism.* Pp. vii., 313. Leipzig: The University Press, Limited. Philadelphia: The F. A. Davis Company. 1900.

Some interest out of the ordinary attaches to this work, because of an unwise prosecution of a bookseller in London by the Commissioners of Police for the sale of vol. i., which it was alleged was issued with the intent to corrupt the morals of the people. The second volume, therefore, was published abroad.

There can be no doubt, we think, as to the unadvisability of the issue of this and kindred works, some of which are translations from foreign tongues into English, without any restriction whatever on their sale. They are certainly stated to be issued for scientific purposes only, and to no one but medical men, teachers, lawyers, and the clergy (a very wide *clièntele*); but it is a well-known fact that they can be easily obtained by any and every one, and are being so obtained for the satisfaction of a prurient and unhealthy curiosity. Far be it from us to say that such works should not be published, but it would certainly seem extremely desirable for the good of our younger generation that a wholesome restriction of a more stringent sort than at present exists should be exercised over their sale now that the good old practice of writing such works in the Latin tongue, through decay of classical knowledge, has ceased to exist.

The publication of the translation of Dr. R. von Krafft-Ebing's *Psychopathia Sexualis*, and of Dr. Tarnowsky's work and several others, has led to a great deal being known generally of the strange power which sexuality exercises over the feelings, thoughts, and conduct of men and women at large. The subject, though for long past well known to those engaged in psychological work, and on its darkest side to the medical jurist, has not perhaps received that attention which it ought to have done at the hands of medical practitioners. Many of the strange ideas of patients; many of the unaccountable actions of the young; numerous morbid fancies, or unusual contraventions of the ways of decent society, might have been accounted for satisfactorily by the physician if more had been known of the anomalies of the sexual instinct; and if it had been realised that many of these acts in their more outrageous and criminal forms were certainly symptoms of a constitutional malady, or of a weakness of cerebral condition thus manifesting itself.

Now that men and women, through these works and in other ways, have eaten of the tree of the knowledge of good and evil, it seems necessary that medical men should obtain some exact knowledge of the causes and conditions of the development of these sexual aberrations, and how they may be the product of, or be influenced by, hereditary constitution, education, and modern conditions of society, as well as of the methods by which they should be treated. For this purpose some study of the evolution of man, of love, the married life, and the development of the sexual anomalies must be acquired. In Mr. Havelock Ellis's book we find much that is useful to this end set forth clearly and in a fairly scientific way. The chapters on the Evolution of Modesty, and the Phenomena of Sexual Periodicity in vol. ii. are particularly interesting. We observe that little importance seems to be attached to the religious side of the question, or the great help that may be obtained in the treatment of this class of case by an appreciation of the duty of self-restraint and chastity imposed upon us as Christians. The work is worthy of attention and consideration, though assent cannot be given to all the deductions of the author.

